# Reconstruction of larval origins based on genetic relatedness and biophysical modeling

**DOI:** 10.1038/s41598-019-43435-9

**Published:** 2019-05-08

**Authors:** I. Segura-García, L. Garavelli, M. Tringali, T. Matthews, L. M. Chérubin, J. Hunt, S. J. Box

**Affiliations:** 1 National Museum of Natural History-Smithsonian Marine Station at Fort Pierce, 701 Seaway Dr, Fort Pierce, 34949 FL USA; 2 Pacific Northwest National Laboratory, 1100 Dexter Ave N, Suite 500, Seattle, WA USA; 3 Florida Fish and Wildlife Conservation Commission, Fish and Wildlife Research Institute, 100 Eighth Avenue S.E., St. Petersburg, 33701 FL USA; 4 Florida Fish and Wildlife Conservation Commission, Fish and Wildlife Research Institute, 2796 Overseas Hwy. Suite 119, Marathon, 33050 FL USA; 5 Florida Atlantic University-Harbor Branch Oceanographic Institution, 5600 US Highway 1 North, Fort Pierce, 34946 FL USA; 6 Rare, 1310N, Courthouse Road, Suite. 110, Arlington, 22201 VA USA

**Keywords:** Molecular ecology, Population dynamics

## Abstract

The assessment of the mechanisms and patterns of larval connectivity between geographically separated populations leads to a better understanding of benthic marine population dynamics, especially in commercially valuable species. This study investigated for the first time the fine-scale temporal genetic variability of new settlers and their origins in a benthic marine organism with one of the longest pelagic larval phases, the Caribbean spiny lobster (*Panulirus argus*). We genotyped newly settled postlarvae in the Florida Keys and adults of spiny lobster from the Florida Keys and throughout the Caribbean Sea. We identified strong larval connectivity between Dominican Republic, Belize, Nicaragua, the Florida Keys, and West-Florida. The larval dispersal modeling suggests that Florida’s lobster population could receive recruits from within and from other areas outside its state and national maritime boundaries. The genetic analyses refine the oceanographic model indicating that the connectivity patterns described could also result from unknown parental populations sourcing adults and postlarvae in different spawning seasons to the Florida Keys. We discuss the importance of small temporal scales to identify patterns in larval export. Our findings are significant on two levels. From the larval dispersal perspective, genetic results and biophysical modeling identify patterns of gene flow enhancing persistence of local populations. From an economic and fishery perspective, *P*. *argus* is the most important commercial species in the Caribbean and our results inform how considering larval source and sink dynamics across international boundaries could improve management plans at local, national, and regional levels.

## Introduction

The increasing number of fisheries categorized as fully or overexploited^1,2^ demonstrates the need to more fully understand population dynamics of key fisheries to prevent or reverse stock decline and collapse. Fisheries data are used to inform how much is removed from the ecosystem, but knowing how much is recruited, the origin of those recruits, and the spatiotemporal variability in those patterns is further information required to assess population dynamics and to determine the suitable spatial scales to manage fished populations. This is especially critical for commercially important species with a broad distribution, complex life cycles, and that suffer high removal rates through chronic fishing pressure^3,4^.

The Caribbean spiny lobster, *Panulirus argus* (Latreille, 1804), occurs throughout the Caribbean basin and north to Bermuda and is the most important commercial fishery in the Caribbean Sea^5,6^. Amongst marine invertebrates, *P*. *argus* has one of the longest larval dispersal phase, lasting up to nine months in the plankton^7,8^. Spiny lobster planktonic larvae have limited horizontal swimming ability and are therefore subject to widespread dispersal by currents^9^, but also undertake ontogenetic vertical migration during their dispersal phase^10^. The final larval stage metamorphoses into a non-feeding postlarva that actively swims from oceanic waters to the coast wherein they settle in shallow protective habitats^9^. The complex pelagic phase in the life cycle of *P*. *argus* has made it a species difficult to study for population dynamics.

Larval dispersal knowledge has benefited from the use of direct genetic methods, which have provided new evidence of different patterns of local retention, self-recruitment, or asymmetrical gene flow in marine organisms (e.g. reef fishes^11–13^, pink abalone^14^, California red rock lobster^15^, and demersal fish^16^). Nonetheless, larval transport and recruitment are known to be affected by the variability of oceanographic currents and biological factors, such as the larval dispersal duration, spawning abundance and characteristics, and larval behaviours. Hence, complicating the full assessment of benthic population dynamics. For example, in the emperor fish, *Lethrinus nebulosus*, extensive gene flow was predicted in biophysical modelling of passive particles, however, common history of spawning and cohesiveness of larval transport pathways resulted in a non-random genetic structure^17^. In *P*. *argus*, the predicted average distance a larva settles from its release location is reduced by over 60% when larval diel vertical migration is incorporated into a biophysical transport model^10^. Another factor affecting the assessment of larval dispersal is the effect of climate change; in the European lobster, *Homarus gammarus*, the increase of water temperature is expected to increase temperature-dependent mortality of larvae, as a result of a forward shift in hatching and poor quality and abundance of food^18^. The effects of climate change on the larval development and therefore recruitment to fishery strongly depend on the biology and phenology of the species and its vulnerability to environmental stressors^19^.

Previous studies have contributed to the understanding of spiny lobster postlarvae recruitment and connectivity, suggesting that local populations depend on asymmetric larval supply from many potential source regions across the Caribbean but also identifying that self-recruitment occurs^20–25^. However, the direct genetic comparison between adults and recruits has yet to be investigated. In this study, we used both indirect and direct genetic methods to investigate the fine-scale temporal variation in larval connectivity and recruitment by estimating the extent of relatedness between postlarvae settled in the Florida Keys and adult spiny lobsters from geographically remote populations across the Caribbean Sea. Indirect oceanographic modeling methods were used to investigate the transport and retention patterns of spiny lobster larvae in the Caribbean region. The modelling results were compared with the genetic results.

## Results

### Genotyping

A total of 2030 postlarvae, from the lower and middle Florida Keys, and 799 adult spiny lobsters, from 15 locations throughout Florida and the Caribbean Sea, were genotyped for 14 microsatellite loci. Departures from Hardy-Weinberg equilibrium (HWE) were consistent in both adult and postlarvae databases for several loci, thus genetic diversity estimates for combined postlarvae and adult data were estimated and summarized (Table S1). All loci were retained for further analyses given the high relatedness resolving power estimated by the polymorphic information content (PIC), (Table S1).

### Temporal genetic differentiation

**Figure 1:**
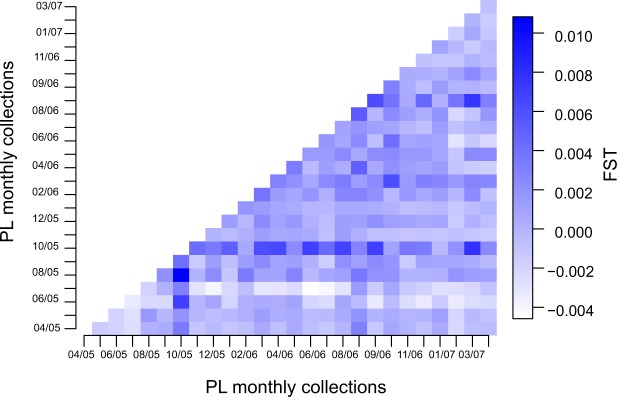
Heatmap of pairwise *F*_*ST*_ values estimated from microsatellite data between all monthly *Panulirus argus* postalarvae collections. This illustrates the complex temporal genetic variation of settlers in the Florida Keys. The darker color indicate stronger genetic differentiation.

**Figure 2:**
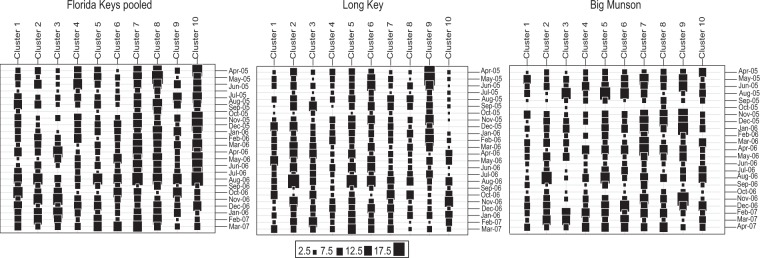
Cluster analysis as implemented in DAPC of microsatellite loci genotypes of *Panulirus argus* postlarvae collected in the Florida Keys (two sites pooled), Long Key (middle Florida Keys) and Big Munson (lower Florida Keys). The proportional contribution of each genotypically described cluster in each monthly sample is indicated by the size of the black boxes.

Genetic differentiation of postlarvae monthly samples was overall low, *F*_*ST*_ values ranged from −0.004 to 0.0079, but showed a complex pattern of genetic differentiation where some months were slightly less differentiated than others (Fig. 1). The DAPC cluster analysis suggested an optimum of 10 genetic clusters describing the postlarvae genotype variability, while individual postlarvae from each monthly sample were assigned in different proportions to each of these clusters throughout the time of the study (Fig. 2).

### Postlarvae source populations

**Figure 3:**
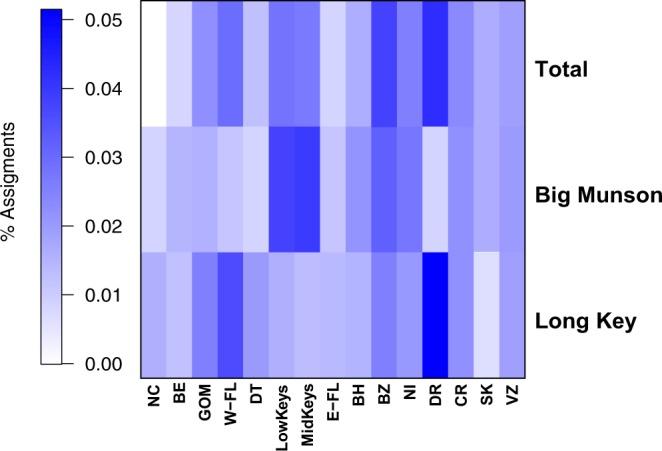
Heat-map of the weighted percentage of assignments of *Panulirus argus* postlarva to adult population as implemented in the parentage analysis in CERVUS. Acronyms of adult populations are: NC = North Carolina, BE = Bermuda, GOM = Gulf of Mexico, W-FL = Western Florida, Low-FK = Lower Florida Keys, Mid-FK = Middle Florida Keys, E-FL = Eastern Florida, BH = Bahamas, BZ = Belize, NI = Nicaragua, DR = Dominica Republic, CR = Saint Croix, SK = Saint Kitts, VZ = Venezuela. The percentage of postlarvae assigned to parental populations was weighted by adult sample size and total number of postlarvae collected.

The parentage assignment test for all 2030 postlarvae was resolved for 226 individual postlarvae (11%); of these, 28.3% (64 individuals, 3% of the total postlarvae), were assigned to the Florida Keys adult population (Table 1). The weighted proportion of these assignments to each potential parental population (i.e. adult samples included in this study) indicated postlarvae settlers had a strong connectivity with adult populations from the Dominican Republic (0.0514), Belize (0.0406), West Florida (0.0362), and Nicaragua (0.0313); but also to local adult populations such as Lower Keys and Middle Keys (0.0346 and 0.0327, respectively) (Fig. 3).

**Table 1:** Summary of number of *Panulirus argus* recruits assigned to each source-population (parentage assignment) as estimated in CERVUS.

		NC	BE	GOM	W-FL	DT	lFK	mFK	E-FL	BH	BZ	NI	DR	CR	SK	VZ
N adult		40	49	16	39	50	119	77	49	54	48	22	36	102	49	49
Assignments	Long key	5	4	4	15	9	15	7	5	6	12	4	21	21	0	8
	Big Munson	0	2	0	6	0	25	17	1	5	8	3	0	10	9	4
%	Long key	0.4409	0.3527	0.3527	1.3228	0.7937	1.3228	0.6173	0.4409	0.5291	1.0582	0.3527	1.8519	1.8519	0.0000	0.7055
	Big Munson	0.0000	0.2232	0.0882	0.0882	0.0000	2.7902	1.8973	0.1116	0.5580	0.8929	0.3348	0.0000	1.1161	1.0045	0.446429
weighted %	Long key	0.0110	0.0072	0.0220	0.0339	0.0159	0.0111	0.0080	0.0090	0.0098	0.0220	0.0160	0.0514	0.0182	0.0000	0.0144
	Big Munson	0.0000	0.0046	0.0055	0.0023	0.0000	0.0234	0.0246	0.0023	0.0103	0.0186	0.0152	0.0000	0.0109	0.0205	0.0091
	total	0.0110	0.0118	0.0276	0.0362	0.0159	0.0346	0.0327	0.0113	0.0201	0.0406	0.0313	0.0514	0.0291	0.0205	0.0235

Adult population as follow: NC = North Carolina, BE = Bermuda, GOM = Gulf of Mexico, W-FL = Western Florida, lFK = Lower Florida Keys, mFK = Middle Florida Key, E-FL = Eastern Florida, BH = Bahamas, BZ = Belize, NI = Nicaragua, DR = Dominican Republic, CR = Saint Croix, SK-Saint Kitts, VZ = Venezuela. Adult sample size at each location: N adult, number of postlarvae assigned to each adult population: Assignments, percentage of postlarvae assigned to each location: %, and percentage of postlarvae assigned to each location weighted by adult sample size: weighted %.

### Larval dispersal model

**Figure 4:**
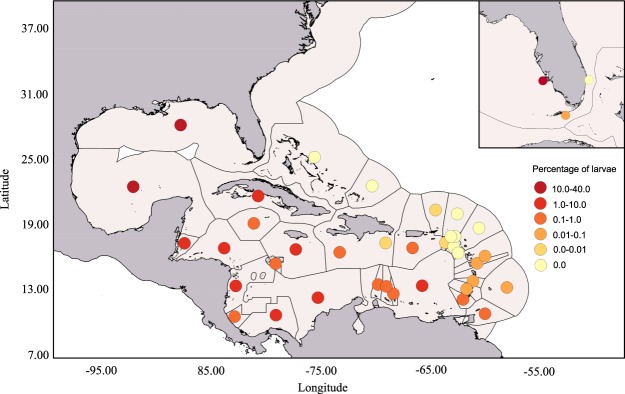
Distribution of *Panulirus argus* larvae after 196 days of simulated backward transport in the Caribbean region using the low resolution model (HYCOM, horizontal resolution 8 km). Virtual larvae were released from two locations in the Florida Keys: Long Key (middle Florida Keys) and Big Munson (lower Florida Keys). See Fig. 6 for the location of the release polygons (black arrows). The percentage of larvae was averaged in each Exclusive Economic Zone of the Caribbean region and are shown as heat colored circles.

**Figure 5:**
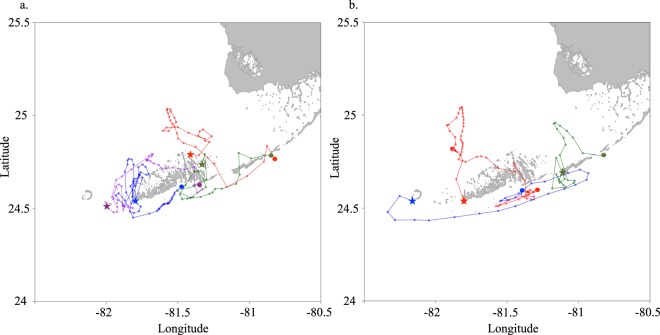
Examples of larval trajectories of *Panulirus argus* during the simulated backward transport from settlement locations using the high resolution model (ROMS, horizontal resolution ~2.8 km). Virtual larvae were released in September 2007 (**a**) and October 2007. (**b**) The predicted origin location of each larva is marked with a star (i.e. release) and the circle indicates its settlement location.

To investigate the large-scale connectivity, a large-scale Caribbean wide model was used to predict release (spawning) locations for the postlarvae which settled at the two collection sites (Lower Florida Keys: 24.617°N, 81.387°W; Middle Florida Keys: 24.803°N, 80.84°W). The sources of larvae were particularly important in the west coast of Florida, northwest Cuba, the Caribbean coast of Mexico, Belize, and Honduras (Fig. 4). Within the Florida region, the local model predicted the postlarvae that settled in the Lower and Middle Florida Keys mainly originated from habitats near the Keys (see Fig. S1 in Supporting Information). Trajectories of these larvae that originated and settled in the Keys showed two main paths: northward and southward the Keys (e.g. Fig. 5). The releasing locations of the larvae were consistently located west of their settlement locations.

## Discussion

The present study is the first to investigate fine-scale genetic relatedness between adults and new settlers of spiny lobster using a parentage analysis as a proxy to assess the reproductive connectivity of postlarvae in the Florida Keys with adult populations across the wider Caribbean.

The parental assignment based on the extent of alleles shared between Florida Keys postlarvae and adult spiny lobsters, indicated a high degree of connectivity to Dominican Republic, Belize, Nicaragua, West Florida and the Florida Keys. The backward larval dispersal modeling conducted at both Caribbean wide and Florida spatial scales predicted comparable results to the genetic analyses performed. Larval dispersal between the Florida Keys and Belize, Western Florida, Gulf of Mexico, and within the Florida Keys itself was indeed predicted by the models. These results are overall consistent with the major larval exporter countries previously suggested by Kough *et al*.^23^ (i.e. Venezuela, Nicaragua, Belize and Dominican Republic). Although including all the potential sources of postlarvae in our analysis could better resolve the larval origins and connectivity of the Florida Keys recruits, it remains difficult to achieve given the broad distribution of the species.

Previous modeling studies have suggested that larval recruitment in the northern regions of the Caribbean are highly dependent on distant sources supplied by strong northward currents, while recruitment dynamics in the southern regions of the Caribbean are more influenced by retentive oceanographic structures, dominated by persistent gyres^10,20^. In this regard, high connectivity between Florida, southwest Cuba, and the Mexican Caribbean might be expected considering the proximity of these sites to the Yucatan Current where advection is likely to occur despite of the occurrence of persisting recirculation observed in southwest Cuba and the Campeche Bank (see Briones-Fourzán *et al*.^20^). Similarly, Zeng *et al*.’s^26^ dispersal model for bonefish larvae (53 days of pelagic phase duration) showed high connectivity between Belize, Mexico, Cuba, and the Florida Keys, but also showed that the Lower and Middle Key have the highest retention rate as seen in our local model. In a recent study, Garavelli *et al*.^27^ showed that the highest values of local retention and self-recruitment in the Caribbean wide region were observed in Mexico and Florida. These high retention rates were driven by the availability of favorable habitat and the populations in each region were found self-persistent, meaning that they did not rely on external larval supply to maintain their population^27^. Therefore, despite potential significant influx from other geographically close sources, the estimated percentage of postlarvae assigned to the Florida Keys could remain the same.

Along the Florida Keys the coastal counter-current and the Tortugas Gyre, when present, could play an important role in the spiny lobster postlarvae transport, survival, and recruitment^28,29^. The strong genetic relatedness between postlarvae and adults from the Florida Keys supports the hypothesis that the southwest Florida shelf area plays an important role in the local retention and self-recruitment of larvae originated in the Florida Keys. The local retention and self-recruitment are also assisted by elevated nutrient levels from the upwelling in the interior and fronts of the eddies and gyres that surround the Florida Keys^29,30^. This upwelling enhances larval food supply and potentially may increase larval survival^28^. The Florida regional model simulation confirmed the retention role of the southwest Florida shelf (Fig. 5), with postlarvae coming from both the northern and southern parts of the Keys and predicted drift patterns similar to drifter trajectories in the same region shown in Yeung *et al*.^29^. This result is also consistent with the recent observations describing the southward movement of reproductively active females in the Keys^31^ that would spawn within the counter current area. Lee and Williams^32^ identified several “recruitment conveyors” forming a network of retention pools (gyres) on both sides of the Florida Keys (Fig. S2). The longest retention pool associated with a location that includes adult spiny lobsters has a 6–8 months retention time and is located north of the Dry Tortugas. But Lee and Williams^32^ also showed the likeliness of offshore spawned larvae to be transported to the coastal zone and channels between the Keys by the bottom Ekman transport^29^. Lobster larvae can then migrate to the retention pool on the south Florida shelf. In addition, larvae could be released on the Southwest Florida Shelf north of the Florida Keys.

More exhaustive genetic analysis would be needed to allow the differentiation between parent-offspring relationships from sibling relationships from multiple year classes. Therefore, the most parsimonious explanation for our results is that a same parent population was the source of adults and postlarvae sampled in the locations in the Florida Keys across different spawning seasons. Indeed, the low levels of relatedness to adult populations downstream from the Florida Keys in East Florida (0.0113), North Carolina (0.0110), and Bermuda (0.0118) support this hypothesis, most likely due to the lack of significant reverse flow near the Gulf Stream, which is incompatible with the concept of a parental relationship.

Self-recruitment has also been suggested in a congeneric species, *P*. *interruptus*, in the Mexican Pacific coast where a high proportion of kinship among adult lobsters was found^15^. However, for *P*. *argus* in Florida, the strong spawner *vs*. recruit correlation suggestive of self-recruitment^33^, was reassessed and patterns observed seem to represent broader adult abundance from Caribbean wide origin^34^. Thus, the contribution of local Florida spawning stock to local post-larval recruitment remains unresolved by using adult lobster abundance and postlarvae recruitment patterns alone.

The use of direct genetic methods at fine temporal scales allowed the identification of complex genetic variability in recruits (Table 1, Fig. S3) yielded by asymmetrical gene flow, where some locations (e.g. Florida Keys, Belize and US Virgin Islands) consistently provide disproportionately more larvae to the Florida Keys than other populations during the time of the study (Fig. S4). In the rock lobster, *P*. *cygnus*, the combination of temporal genetic variation and dissimilar recruitment patterns at two sites resulted in genetically different cohorts, but was also different from following time replicates^35^. Similarly, our results from the DAPC clustering analyses showed temporal variation of the inferred clusters and that clusters 7–10 were more represented than cluster 1–6 throughout the two years of the study (Fig. 2). This temporal genetic variability is normally neglected using indirect genetic analyses, as previously estimated between *P*. *argus* adult populations^36,37^ and in this study (Fig. S3), but is shown here to contribute to population dynamics and structuring. Our results provide evidence supporting the hypothesis that spiny lobster populations are not as vagrant and assorted as previously thought, as indicated by the low but significant levels of genetic differentiation between some populations in Central America^38^, and the significant differences found between geographically close basins but not between most distant basins^25^. Likewise, across the range of the American lobster, *Homarus americanus*, a hierarchical genetic structure revealed significant differences between north (Gulf of St. Lawrence) and south (Gulf of Maine) regions and among populations within these regions^39^.

In marine invertebrates with pelagic larval phase, *F*_*ST*_ values are typically very low (<0.05), which challenges the inference of genetic structure^40^. The low genetic differentiation as estimated by *F*_*ST*_ may have resulted from significant gene flow or larval connectivity over evolutionary time scales that homogenize allele frequencies but, does not necessarily mean that populations are well mixed on ecological time scales. This supports the hypothesis that stepping-stone import of foreign genotypes will result in panmixia over an evolutionary time scale^41^. However, to correctly describe the ecological processes at work to inform management decision making, it is crucial to detect the fine-scale temporal variability of settlement and identify the consistent and key sources of recruits into local populations which may vary through time.

Population modeling studies on *P*. *argus* in Honduras suggested that a reserve network at country scale will result in a significant increase of both persistence and yield of the species^42^. This is consistent with the hypothesis that Caribbean spiny lobster populations are influenced by both open and closed recruitment dynamics. Thus, management actions at national level to conserve spawning stock biomass may deliver improvements at a country scale given the correct oceanographic conditions even for species with long pelagic larval phases. Including the study of genetic variability at these shorter temporal and finer spatial scales is key to elucidating patterns in larval export and determining the importance of self-recruitment which may otherwise be masked in larger scale studies. Understanding these patterns can help assess the potential contribution from different levels of management intervention, from local to international, for marine species with a broad distribution and complex dispersal patterns.

## Methods

### Sample collection

**Figure 6:**
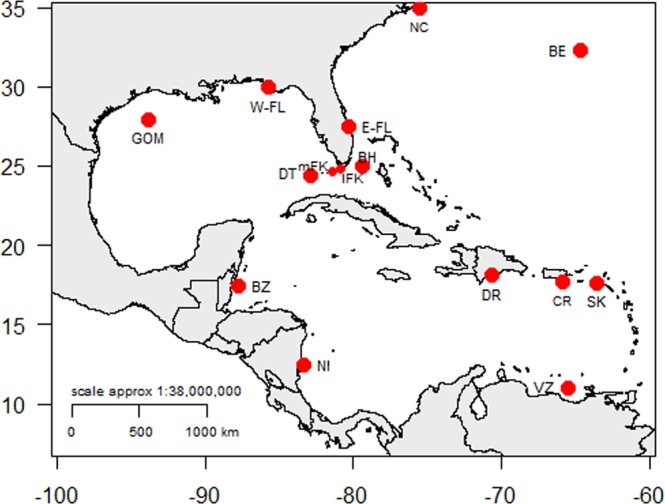
Map of the Caribbean basin showing sampling locations of adult lobsters *Panulirus argus*. NC = North Carolina, BE = Bermuda, GOM = Gulf of Mexico, W-FL = Western Florida, DT = Dry Tortugas, lFK = Lower Florida Keys, mFK = Middle Florida Keys, E-FL = Eastern Florida, BH = Bahamas, BZ = Belize, NI = Nicaragua, DR = Dominican Republic, CR = Saint Croix, SK-Saint Kitts, VZ = Venezuela.

From April 2005 to March 2007, postlarvae from Florida were collected on the seventh day of each lunar month (*n* = 50 targeted per lunar month), using five artificial collectors placed parallel to the shore near inter-island channels and anchored near shore in shallow waters (<2 m) from two long-term monitoring sites near Big Munson, in the Lower Florida Keys (24.617°N, 81.387°W), and the south side of Long Key, in the Middle Keys (24.803°N, 80.84°W) (Fig. 6). Whole individual postlarvae were preserved into vials containing 90% ethanol and were identified by sample location, date, and individual identification number.

We collected tissues from adult spiny lobsters from 15 sites throughout the U.S. and Caribbean regions including North Carolina, the northernmost limit of species distribution, the Gulf of Mexico Flower Gardens Banks, the Dry Tortugas, Florida Lower Keys, the Upper Keys, Fort Pierce (East Florida), Panama City (West Florida), Bermuda, the Bahamas, Belize, Nicaragua, St. Kitts, St. Croix (U.S.Virgin Islands), the Dominican Republic, and Venezuela (Fig. 6). Approximately fifty specimens were collected from each site. Adult samples were collected from harvested individuals. Tissue was obtained from the last segment of a middle walking leg and was excised with sterile scissors. Depending on field conditions, the entire segment or dissected muscle was preserved in 90% ethanol.

### Microsatellite genotyping

Total genomic DNA was extracted and purified from tissue samples using PUREGENE® DNA Isolation Kit (Gentra Systems, Inc., Minneapolis, Minnesota) following manufacturer guidelines. Individual lobsters (postlarvae and adults) were genotyped for 14 bi-parentally inherited microsatellite DNA markers developed for this species^43,44^. PCR conditions were as follow: 94 °C for 2 min, 32 cycles of 94 °C for 40 s, 58 °C for 40 s, 72 °C for 45 s, followed by 72 °C for 7 min. PCR products were visualized on an ABI 3130 genetic analyser and genotyped by using GeneMapper® software v3.7 (Applied Biosystems, Inc.). Genotypes across all 14 microsatellite loci were tested for the presence of allelic dropout and null alleles using the program MICRO-CHECKER^45^. Genetic diversity within each location and monthly collections were assessed using standard measures (number of alleles and allelic richness) estimated in FSTAT 2.9.3^46^. Observed heterozygosity (Ho), expected heterozygosity (He), departures from Hardy-Weinberg equilibrium (HWE) and the power of resolution of the microsatellite loci (PIC) were examined in CERVUS 3.0.7^47^.

### Genetic differentiation among recruits (indirect method)

Temporal genetic differentiation among monthly postlarvae samples was examined based on differences in allele frequency to estimate convectional pairwise *F*_*ST*_ using ARLEQUIN 3.5^48^. Pairwise genetic differentiation was also estimated between postlarvae and adult population samples using convectional pairwise *F*_*ST*_ using ARLEQUIN 3.5^48^. To determine genetic differentiation between postlarvae samples, we used a discriminant analysis of principal components (DAPC)^49^, a multivariate method implemented in the *adegenet* package^50^ for R^51^. The postlarvae collection date was used as a prior. The DAPC approach proposes a distribution of postlarvae into predefined groups in relation to the discriminant function of principal components. The optimum number of clusters was defined by *k*-means algorithm that uses the Bayesian information criterion. This approach is preferred for species exhibiting potentially high gene flow, as DAPC maximizes genetic separation among groups and minimizes variation within groups^49^.

### Inferring larval connectivity (direct method)

A single-parent parentage analysis was conducted to investigate the extent of connectivity between postlarvae settled in the Florida Keys and adult lobsters from other locations, both local within Florida and from other countries internationally. This direct method compared multi-loci genotypes of adults with multi-loci postlarvae genotypes to assign individual postlarvae to a candidate parent. This analysis is a proxy to infer the potential sourcing population, rather than an actual parentage analyses to assign postlarvae to parent populations (recruit-spawner). Given that lobsters are long-lived animals, there is potential for generation overlap that might make it difficult to distinguish between parent-offspring and siblings from a different year class. The parentage analysis was conducted as implemented in CERVUS 3.0.7^47^. The probability for the most likely parent for each postlarva was defined by taking the natural log (log base e) of the overall likelihood ratio (LOD scores). The critical LOD value was determined with a 95% confidence level running parentage simulations for 10,000 offspring while considering 98 candidate parents (half of the adult sample size collected in the Florida Keys) and a proportion of candidate mothers estimated at 0.016, based on the female spawning stock assessment for the Florida Keys in 2004^33^. To increase the strictness of the parentage analyses, we only considered genotype comparisons of more than 10 microsatellite loci and a maximum two pair loci mismatched. The critical LOD score estimated (LOD = 2.5) was the cut-off for assigning the single parent, which is a conservative approach that accounts for some unsampled putative parents in the population^52^. The proportion of postlarvae assigned to different source populations were weighted by the total number of postlarvae and the corresponding adult sample size.

### Larval dispersal model

To estimate the spawning locations of *P*. *argus* larvae settling in the Florida Keys, we modeled the larval dispersal of the species using the offline Lagrangian tool Ichthyop v3.2^53^. Ichthyop v3.2 allows the coupling between a hydrodynamic model and an individual-based model and is based on an Euler advection scheme. In the model, each particle represents a virtual larva and is characterized by its longitude, latitude, and depth in three dimensions. Two simulations were performed: the first one to investigate the transport patterns at large spatial scale in the Caribbean region and the second to investigate the retention patterns around Florida by using a higher spatial resolution ocean model. The large scale simulation used the 8-km horizontal resolution HYCOM consortium global model^54^. The HYCOM model fields were extracted daily for the intra America Seas region and converted to Ichthyop’s input format. At the Florida scale, we used the Regional Oceanic Modeling System (ROMS) high-resolution (~2.8 km) simulation of the south Florida shelf, which includes tides and is described in Criales *et al*.^55^. Two polygons, encompassing the postlarvae collection sites (Lower Florida Keys: 24.617°N, 81.387°W; Middle Florida Keys: 24.803°N, 80.84°W), i.e. settlement locations, were defined. We adapted the size of the polygons depending on the resolution of each model used. In the large-scale model, each polygon was 64 km^2^ and 25 km^2^ in the Florida scale model. Ten thousand particles were released from each polygon at 5 m depth once a month from July to December 2007. The dispersal duration was set to 196 days with 152 days of pre-competency period^8^. An ontogenetic vertical migration behaviour was included in the model following Callwood^56^. The *P*. *argus* virtual larvae were tracked backward in time from their settlement locations to estimate their origin and the percentage of larvae was averaged over the number of polygon per nation in the Caribbean region.

## Supplementary information

Supplementary information

## Data Availability

Microsatellite genotypes generated and analysed during the current study will be available in the DRYAD repository once the manuscript is accepted. 10.5061/dryad.27812c7.
