# Supplementary material for: Reconstruction of larval origins based on genetic relatedness and biophysical modeling
Source: Sci Rep. 2019 May 8;9:7100. doi: 10.1038/s41598-019-43435-9 (PMC6506592; doi:10.1038/s41598-019-43435-9)
Supplement: Supplementary file 1 — Supplementary information [file 41598_2019_43435_MOESM1_ESM.pdf]

Supporting Information

Reconstruction of larval origins based on genetic relatedness and biophysical modeling

Segura-García I, L Garavelli, M Tringali, T Matthews, L Chérubin, J Hunt, S. J Box

Table S1. Summary statistics of 14 microsatellite loci genotyped. NA: number of alleles, Ho: observed heterozygosity, He: expected heterozygosity, HWE: Hardy-Weinberg equilibrium test, NS: not significant, S: significant, PIC: polymorphic information content.

	Postlarvae + Adults				
Locus	N_A	Ho	He	HWE	PIC
Par-FWC04	48	0.923	0.943	NS	0.940
Par-FWC05	13	0.434	0.751	S	0.713
Par-FWC07	35	0.935	0.953	NS	0.951
Par-FWC08	56	0.895	0.963	S	0.962
Par-FWC13	5	0.342	0.559	S	0.485
Par-FWC14a	6	0.469	0.477	NS	0.418
Par-FWC14b	30	0.684	0.933	S	0.928
Par-FWC16	13	0.387	0.480	S	0.452
Par-FWC17	12	0.622	0.654	S	0.625
Par-FWC18	41	0.487	0.943	S	0.940
Argus-1	27	0.893	0.906	S	0.899
Argus-2	12	0.292	0.633	S	0.612
Argus-5	16	0.495	0.701	S	0.660
Argus-9	46	0.885	0.938	NS	0.935

Supporting Information

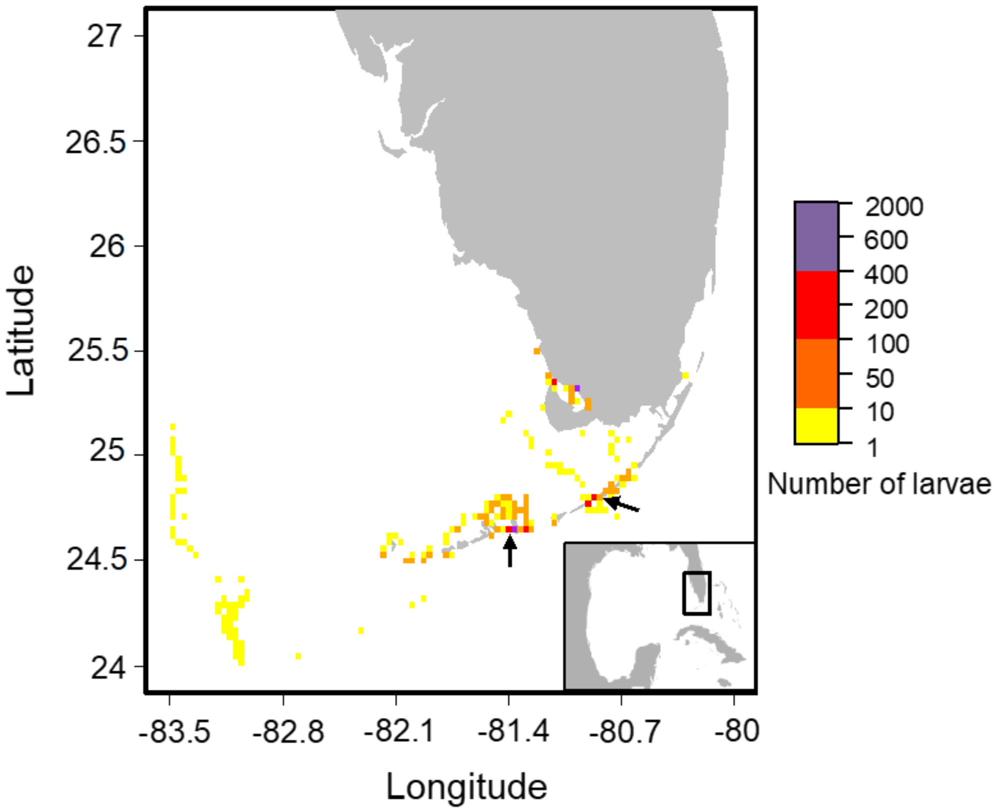

Figure S1. Larval abundance of *Panulirus argus* after 196 days of simulated backward transport around the south Florida shelf using the high-resolution model (ROMS, horizontal resolution ~2.8 km). Virtual larvae were released from two locations in the Florida Keys: Long Key (middle Florida Keys) and Big Munson (lower Florida Keys). The release locations are marked as black arrows on the figure.

Supporting Information

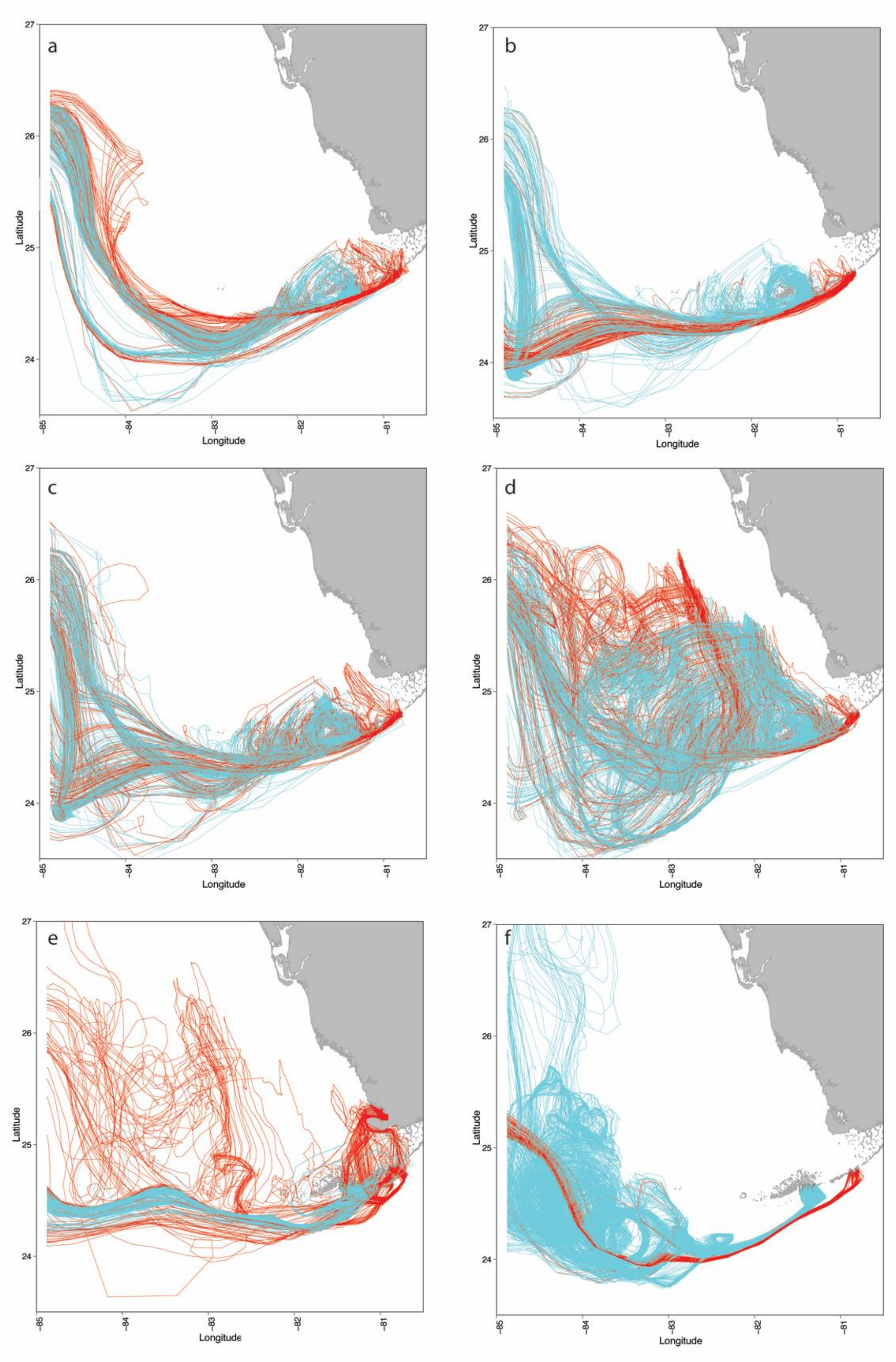

Figure S2: Virtual backward larvae trajectories from the Middle Keys (red) and from the Lower Keys (blue) for the months of July (a), August (b), September(c), October (d), November (e), and December (f). The trajectories are plotted at one-day interval. They reveal the potential origin of the lobster larvae from the west Florida shelf in general and confirm the retention pools suggested by Lee and Williams (1999)

and Yeung et al., (2001), which depends on the position of the Loop Current (LC) and of the presence of eddies on the eastern flank of the LC, and between the Florida Keys and the Florida Current to the south.

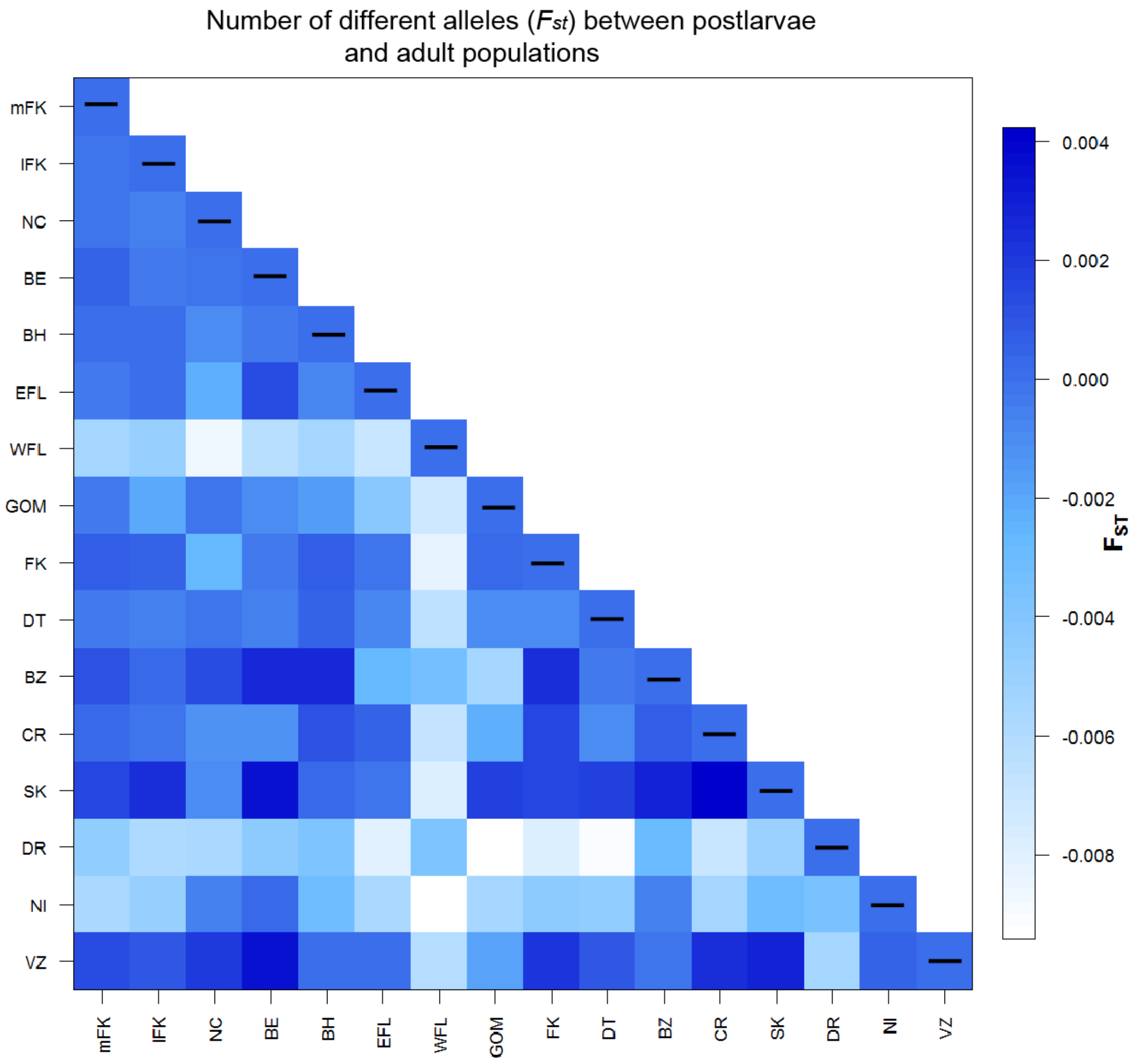

Figure S3. Heatmap of pairwise F_{ST} values color-coded, between each postlarvae samples (mFK, lFK) and each adult population, NC: North Carolina, BE: Bermuda, BH: Bahamas, E-FL: East Florida, W-FL: West Florida, GOM: Gulf of Mexico, FK: Florida Keys, DT= Dry Tortugas, BZ: Belize, CR= Saint Croix, DR: Dominican Republic, NI: Nicaragua, VZ: Venezuela, darker blue indicates more differentiated than lighter colored comparisons.

Supporting Information

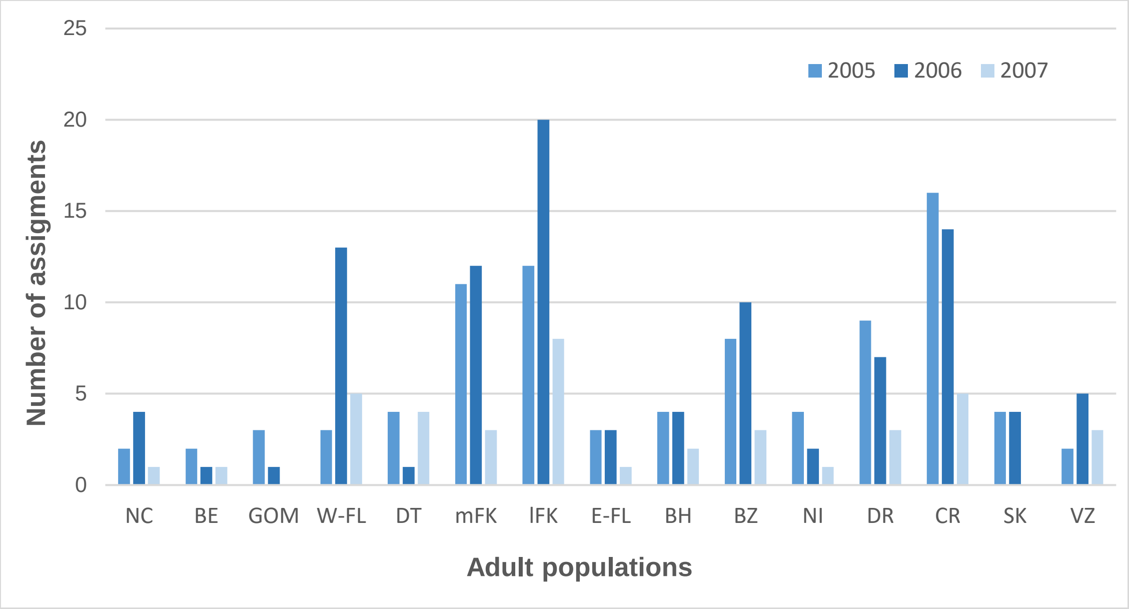

Figure S4. Temporal variation of number of recruits assigned to each adult sample, BE=Bermuda, NC= North Carolina, GOM= Gulf of Mexico, W-FL= West Florida, DT= Dry Tortugas, mFK= Middle Florida Key, lFK= Lower Florida Keys, E-FL= East Florida, BH=Bahamas, BZ= Belize, NI= Nicaragua, DR= Dominican Republic, CR= Saint Croix, SK= Saint Kitts, VZ=Venezuela.
